# Light Emitting Diodes Irradiation Regulates miRNA-877-3p to Promote Cardiomyocyte Proliferation

**DOI:** 10.7150/ijms.70743

**Published:** 2022-07-11

**Authors:** Xinlu Gao, Hanjing Li, Xiuxiu Wang, Zhongyu Ren, Yanan Tian, Jingxuan Zhao, Wenyi Qi, Hongbo Wang, Ying Yu, Rui Gong, Hongyang Chen, Haoyu Ji, Fan Yang, Wenya Ma, Yu Liu

**Affiliations:** 1 Department of Laboratory Medicine at the Fourth Affiliated Hospital, and Department of Pharmacy at the Second Affiliated Hospital, Harbin Medical University, Harbin, China.; 2 Department of Pharmacology (State-Province Key Laboratories of Biomedicine-Pharmaceutics of China, Key Laboratory of Cardiovascular Research, Ministry of Education) at College of Pharmacy, Harbin Medical University, Harbin, China.; 3 Northern Translational Medicine Research and Cooperation Center, Heilongjiang Academy of Medical Sciences, Harbin Medical University, Harbin, China.

**Keywords:** Photobiomodulation, Light-emitting diodes, Cardiomyocyte proliferation, MicroRNA

## Abstract

Mammalian cardiomyocytes (CMs) maintain a low capacity for self-renewal in adulthood, therefore the induction of CMs cycle re-entry is an important approach to promote myocardial repair after injury. Recently, photobiomodulation (PBM) has been used to manipulate physiological activities of various tissues and organs by non-invasive means. Here, we demonstrate that conditioned PBM using light-emitting diodes with a wavelength of 630 nm (LED-Red) was capable of promoting the proliferation of neonatal CMs. Further studies showed that low-power LED-Red affected the expression of miR-877-3p and promoted the proliferation of CMs. In contrast, silencing of miR-877-3p partially abolished the pro-proliferative actions of LED-Red irradiation on CMs. Mechanistically, GADD45g was identified as a downstream target gene of miR-877-3p. Conditioned LED-Red irradiation also inhibited the expression of GADD45g in neonatal CMs. Moreover, GADD45g siRNA reversed the positive effect of LED-Red on the proliferation of neonatal CMs. Taken together, conditioned LED-Red irradiation increased miR-877-3p expression and promoted the proliferation of neonatal CMs by targeting GADD45g. This finding provides a new insight into the role of LED-Red irradiation in neonatal CMs biology and suggests its potential application in myocardial injury repair.

## Introduction

Cardiovascular disease remains the leading cause of death in patients all over the world. The mammalian heart is a terminally differentiated organ that maintains a very low frequency of self-renewal under normal physiological conditions. After myocardial injury, adult CMs cannot be generated to compensate for the loss of necrotic tissue 1. As a result, activated fibroblasts produce a large amount of collagen to fill the deficiency in myocardial tissue, which increases the stiffness of myocardial tissue and affects the normal contraction frequency and amplitude of the myocardium, leading to disorders of cardiac contractile function 2, 3. Recently, a series of studies have shattered the notion that adult mammalian myocardium cannot regenerate after damage 4-6. Increasing evidence suggests that myocardial tissue can gain regenerative potential by targeting certain key signaling pathways that induce CMs to re-enter the cell cycle 7. A few of regenerative strategies, including targeting energy metabolism, cytokines, extracellular matrix and non-coding RNAs to prolong the cell proliferation window or promote cell cycle re-entry, have been revealed 8-13. Hence, targeting CMs proliferation has been suggested as a new strategy to promote myocardial repair after injury.

PBM is increasingly used as a non-invasive medical tool to regulate the vital activities of tissues and organs 14. The light source irradiation devices used for PBM mainly consist of coherent lasers and incoherent semiconductor light-emitting diodes (LEDs) 15. Particularly, LED-based phototherapy is a novel type of non-pharmaceutical therapy with the advantages of non-invasive, painless and convenient 16, 17. By far, LED light from the red and near-infrared spectra has been most widely used in the exploration of disease treatment 18.

Blue and red LEDs have been shown to effective promote the proliferation and differentiation of neural stem cells into astrocytes *in vitro* 19. LEDs can increase ATP production, mitochondrial membrane potential and the activity of cytochrome C oxidase in melanoma cells, thereby promoting cell proliferation 20. In addition, LEDs have shown the capability to promote skin activation and wound healing 21, 22. LED-based PBM has also been widely reported in the modulation of neurological disorders, including Alzheimer's and Parkinson's diseases 23, 24.

Although LEDs exhibit a variety of biological activities on different tissues, the regulatory role and mechanism of LEDs on the proliferation of CMs remained still unclear. Therefore, the purpose of this study was to explore whether LED-Red promotes CMs proliferation and regulates the re-entry of the proliferation cycle. Here we found that conditioned LED-Red exerted a positive effect on neonatal CMs proliferation, and further revealed that miR-877-3p was influenced by PBM and negatively regulated GADD45g, which promoted neonatal CMs proliferation.

## Materials and Methods

### Culture of neonatal mice cardiomyocytes

Neonatal CMs were isolated from 1-3 day old KunMing mice, and cut into small chunks after the hearts was rapidly removed. The tissue was then digested with trypsin (Solarbio, China) at 37 °C. After centrifugation, these cells were resuspended in Dulbecco's modified Eagle's medium (DMEM; Life Technologies, USA) supplemented with 10% fetal bovine serum (FBS; Gibco, USA) and 1% penicillin and streptomycin (Life Technologies, USA), and incubated at 37 °C, 5% CO_2_, 95% air in a humidified incubator for 90 min to allow fibroblasts to attach. Afterwards, cell suspensions were collected and placed in culture dishes with approximately 70%-80% confluence preparation for further dispose.

### Illumination method

The 630 nm LED light sources were arranged into a planar matrix with an area of 10 cm^2^ and placed vertically directly above the cell well plates, and the power density of the light sources was measured using a power density meter (p0023164, Thorlabs, Germany) so that the light power density which finally reached the bottom of the cell well plate was 2.5 mW/cm^2^ with an irradiation time of 10 min. The non-LED group was placed under the same environmental conditions, but conducted light-avoidance treatment.

### Transfection

CMs were transfected with GADD45g siRNA, which was purchased from GENERAL BIOSYSTEMS. Transfections were performed by using Lipofectamine RNAiMAX (Invitrogen, USA) according to the manufacturer's protocols. MiR-877-3p mimics, negative control mimics (NC-mimic), miR-877-3p inhibitor and negative control inhibitor (NC-inhibitor) were purchased from GenePharma (China) and transfected according to the manufacturer's protocol. Overexpression of GADD45g was achieved by plasmid construction, which was purchased from Cyagen Biosciences (China) and pcDNA3.1(+)-GADD45g plasmid construction was obtained from GENERAL BIOSYSTEMS. Transfection of these reagents was performed with Lipofectamine 2000 (Invitrogen, USA). Transfection reagents were discarded 6 h after transfection and replaced with medium containing 10% fetal bovine serum (FBS; Gibco, USA) and 1% penicillin and streptomycin (Life Technologies, USA). According to the experimental requirements, protein was extracted after 12 h irradiation and RNA was collected after 10 min irradiation, respectively.

### Isolation of total RNAs and quantitative real-time PCR (qRT-PCR)

Total RNAs were extracted from CMs by using TRIzol reagent (Invitrogen, USA) according to the manufacturer's protocols. Nanodrop 2000 (Thermo Scientific TM, U.S.A) was applied to identify the quality of total RNAs. The RNA samples were then reverse transcribed to cDNA using the High Capacity cDNA Reverse Transcription Kit (Applied Biosystems, USA) following manufacturer's instructions. SYBR Green PCR Master Mix (Applied Biosystems, USA) was used to perform the quantitative real-time PCR. 18S and U6 were used as controls. The sequences of primers are indicated in Table S1.

### Immunofluorescence assay

CMs were fixed with 4% paraformaldehyde (Solarbio, China) for 15 min at RT. After being washed with PBS, cells were permeabilized with 0.4% Triton X-100 (Solarbio, China) in PBS for 60 min, then blocked with goat serum (Boster, China) for 30 min, and incubated at 4 °C with primary antibodies against phospho-Histone H3 (pH3;1:400; 06-570-AF488; Millipore, USA), α-actinin (1:400; ab108198; Abcam, UK) overnight, and then incubated with Alexa Fluor 488-conjugated or Alexa Fluor 594-conjugated secondary antibody for 1 h. The cells were counterstained with DAPI (1:50; C0065; Solarbio, China) to label the nuclei. The pH3 positive CMs were then evaluated with a confocal laser scanning microscope (FV10i; Olympus, Japan).

### Ethynyl-2deoxyuridine incorporation assay

CMs were incubated with 5-ethynyl-2'-deoxyuridine by using Apollo 567 *in vitro* Kit (Ribobio, China) according to the manufacturer's instructions. Briefly, after treatment with culture or transfection, cells were incubated with 5-ethynyl-2'-deoxyuridine for 12 h and fixed with 4% paraformaldehyde at 37 °C for 15 min. After fixation, the cells were washed 3 times with PBS. Cells were then permeabilized with PBS containing 0.4% Triton X-100 (Solarbio, China) at RT for 60 min and washed 3 times with PBS. Cells were blocked with goat serum (Boster, China) for 30 min and incubated overnight at 4 °C with primary antibody against α-actinin (Abcam, UK), followed by incubation with Alexa Fluor 488-conjugated secondary antibody. Next, the Apollo mixture reagent was added to evaluate DNA synthesis, followed by counterstaining with DAPI. The EdU-positive CMs were then evaluated with a confocal laser scanning microscope (FV10i; Olympus, Japan).

**Western Blot analysis**

The total protein from CMs was extracted by RIPA lysis buffer (Beyotime, China), and BCA assay (Beyotime, China) was performed to quantify the protein concentrations. The equal amounts of protein samples 80 - 100 μg were loaded onto 10% or 12.5% SDS-PAGE gel, and transferred to nitrocellulose membrane (Millipore, USA). The membranes were blocked in 5% skimmed milk, and then reacted overnight with primary antibodies against GADD45g (1:1000; YT1832, Immunoway, USA), NSUN2 (1:1000; 20854-1-AP, proteintech, USA), YTHDF2 (1:1000; 24744-1-AP, proteintech, USA), MTUS1 (1:1000; 13436-1-AP, proteintech, USA), and GAPDH (1:1000; AC033; ABclonal, China) at 4 °C. The membranes were finally incubated with horseradish peroxidase (HRP)-conjugated secondary antibodies for 1 h at RT. Western blot quantitative analysis was performed using Odyssey system (LI-COR Biosciences, USA).

**Dual luciferase reporter assay**

By using TargetScan Mouse algorithm database, we found the presence of miR-877-3p binding site in 3' UTR regions of GADD45g, implying that miR-877-3p can bind to GADD45g and inhibit the biological functions of GADD45g. The GADD45g-mutant constructs were then achieved by mutating the seed regions that binds to the miR-877-3p sequence. GADD45g wild type (WT) and the mutant derivative devoid of the miR-877-3p binding site (GADD45g-mut) were cloned with the downstream of the coding region of the luciferase gene in the psiCHECK-2 luciferase vector. Renilla luciferase was the master reporter. Briefly, CMs were seeded in 24-well plate and either miR-877-3p mimic or its negative control were co-transfected respectively with GADD45g-WT and GADD45g-mut dual-luciferase 3'-UTR vector. The transfection was conducted by using Lipofectamine 2000 (Invitrogen, USA). The cells were harvested 48 h post-transfection. The luciferase activity was measured using a Dual-Luciferase Reporter Assay System (Promega, USA). The relative luciferase activity was normalized to renilla luciferase activity.

### Statistics analysis

Data are expressed as the mean ± SEM. Student's t test was used for comparisons between two groups and one-way analysis of variance followed by Bonferroni corrected post hoc t test was used for multi-group comparisons (version 8.0, GraphPad Software Inc., USA). *P* value < 0.05 was considered statistically significant.

## Results

### LED-Red irradiation promotes cardiomyocyte proliferation

**Figure 1:**
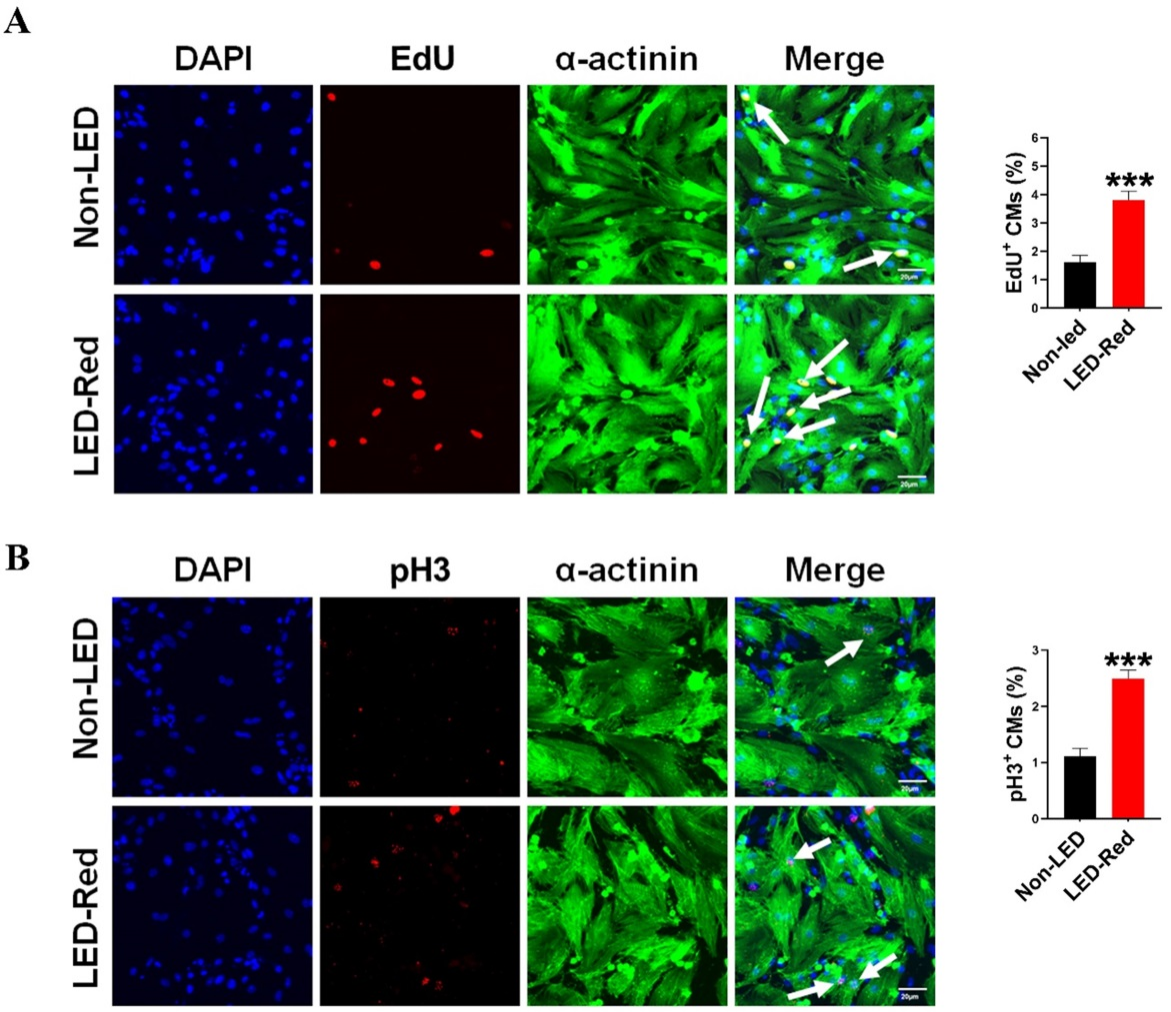
** LED-Red irradiation promotes cardiomyocyte proliferation. (A)** EdU and **(B)** pH3 staining were used to detect the proliferative effect of LED-Red on CMs. The data represent the mean ± SEM. ***P < 0.001 vs. Non-LED. Scale bar = 20 µm.

Although previous studies have demonstrated the ability of PBM to prolong the growth cycle of outer root sheath cells and neural stem cells* in vitro*, the effect of LED-Red on CMs proliferation has not been elucidated yet 25, 26. In a previous study, we found that LED-Red with a power density of 2.5 mW/cm^2^ irradiated for 10 min, could effectively elevating the viability of CMs without phototoxicity on CMs 27. In addition to this, we determined the effect of LED-Red irradiation on the proliferation of neonatal CMs again. The amount of EdU incorporation was measured after neonatal CMs received LED-Red stimulation. As shown in Figure 1A, LED-Red significantly increased the percentage of EdU-positive cells. Furthermore, pH3 staining was employed to observe the effects of LED-Red on mitosis of neonatal CMs. Figure 1B showed that the ratio of proliferating cells labeled with pH3 signal was correspondingly increased after exposure to LED-Red. The above findings confirm that LED-Red irradiation promotes the proliferation of neonatal CMs.

### LED-Red regulates miR-877-3p to promote cardiomyocyte proliferation

**Figure 2:**
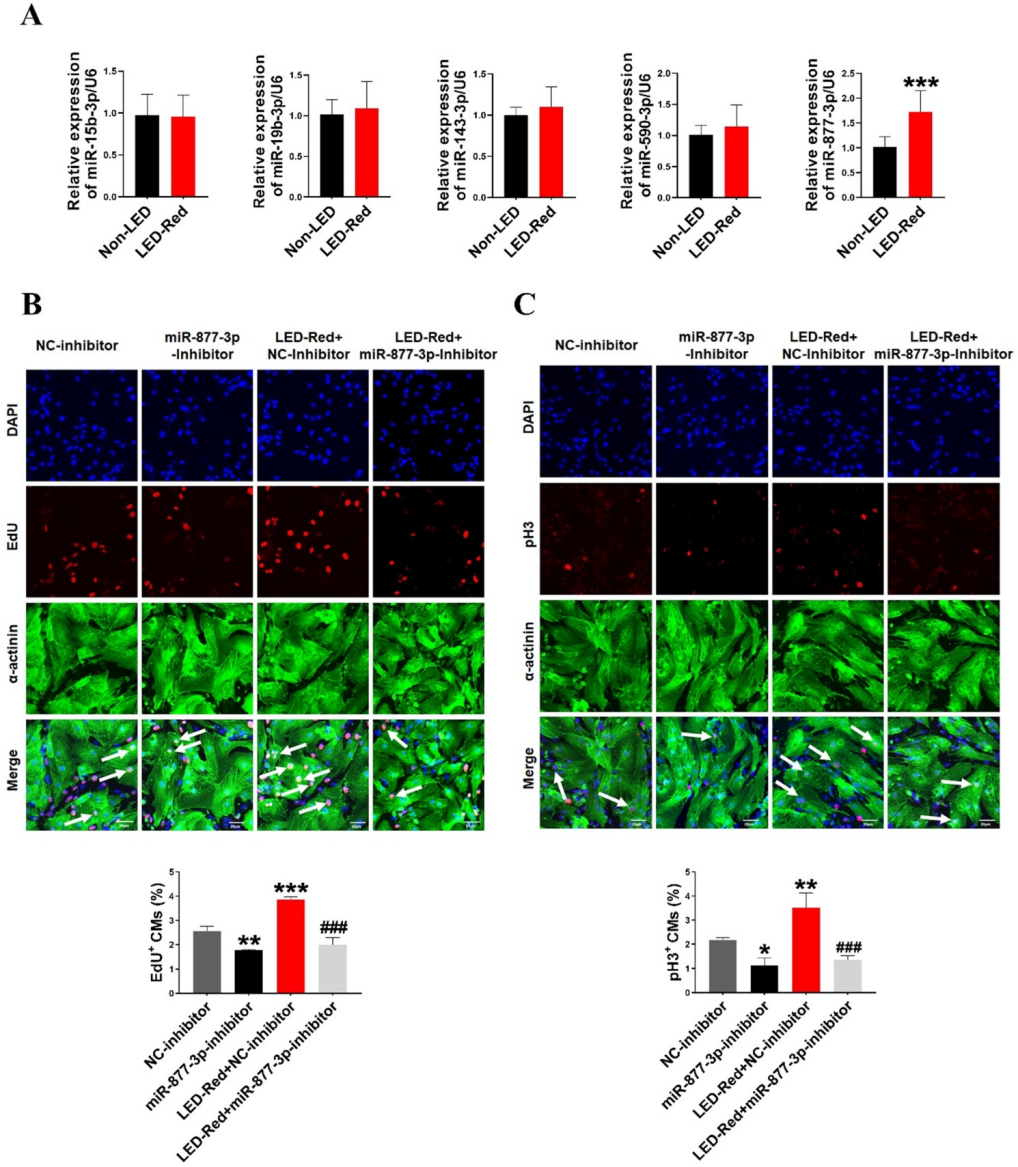
** LED-Red regulates miR-877-3p to promote cardiomyocyte proliferation. (A)** MiRNA expression levels were analyzed by qRT-PCR after LED-Red treatment. **(B)** EdU and **(C)** pH3 staining were performed to examine the effect of co-treatment with miR-877-3p inhibitor and LED-Red on CMs proliferation. Scale bar = 20 µm. The data represent the mean ± SEM *P < 0.05, **P < 0.01, ***P < 0.001 vs. NC-inhibitor, ^###^*P* < 0.001vs. LED-Red + NC-inhibitor group.

Previous researches have shown that miRNAs are essential for the proliferation of CMs 28, 29. Therefore, we next explored the role of miRNAs in the regulation of neonatal CMs proliferation by LED-Red. First, we examined the expression levels of miRNAs after LED-Red stimulation that have been reported to be involved in regulating cardiac diseases or associated with CMs proliferation 28-32. The results showed that LED-Red stimulation significantly up-regulated the expression of miR-877-3p in neonatal CMs, but had no effects on miR-15b and miR-590-3p etc. (Figure 2A). Previous studies have shown that miR-877-3p was involved in apoptosis of CMs 33. However, it remained unclear whether miR-877-3p is engaged in regulating the proliferation ability of CMs. Therefore, we further focused on whether miR-877-3p can regulate neonatal CMs proliferation and whether it was impacted by LED-Red. First, we found that the silencing of miR-877-3p reduced neonatal CMs proliferation, as evidenced by a decrease in the cell proliferation markers EdU and pH3 in the miR-877-3p inhibitor-treated group (Figure 2B, C). More importantly, the results showed that inhibition of miR-877-3p expression impaired the proliferation ability of LED-Red treated neonatal CMs. Collectively, the above results suggest that miR-877-3p serves as a downstream target of LED-Red to regulate the proliferative of neonatal CMs.

### MiR-877-3p regulates cardiomyocyte proliferation through targeting GADD45g

**Figure 3:**
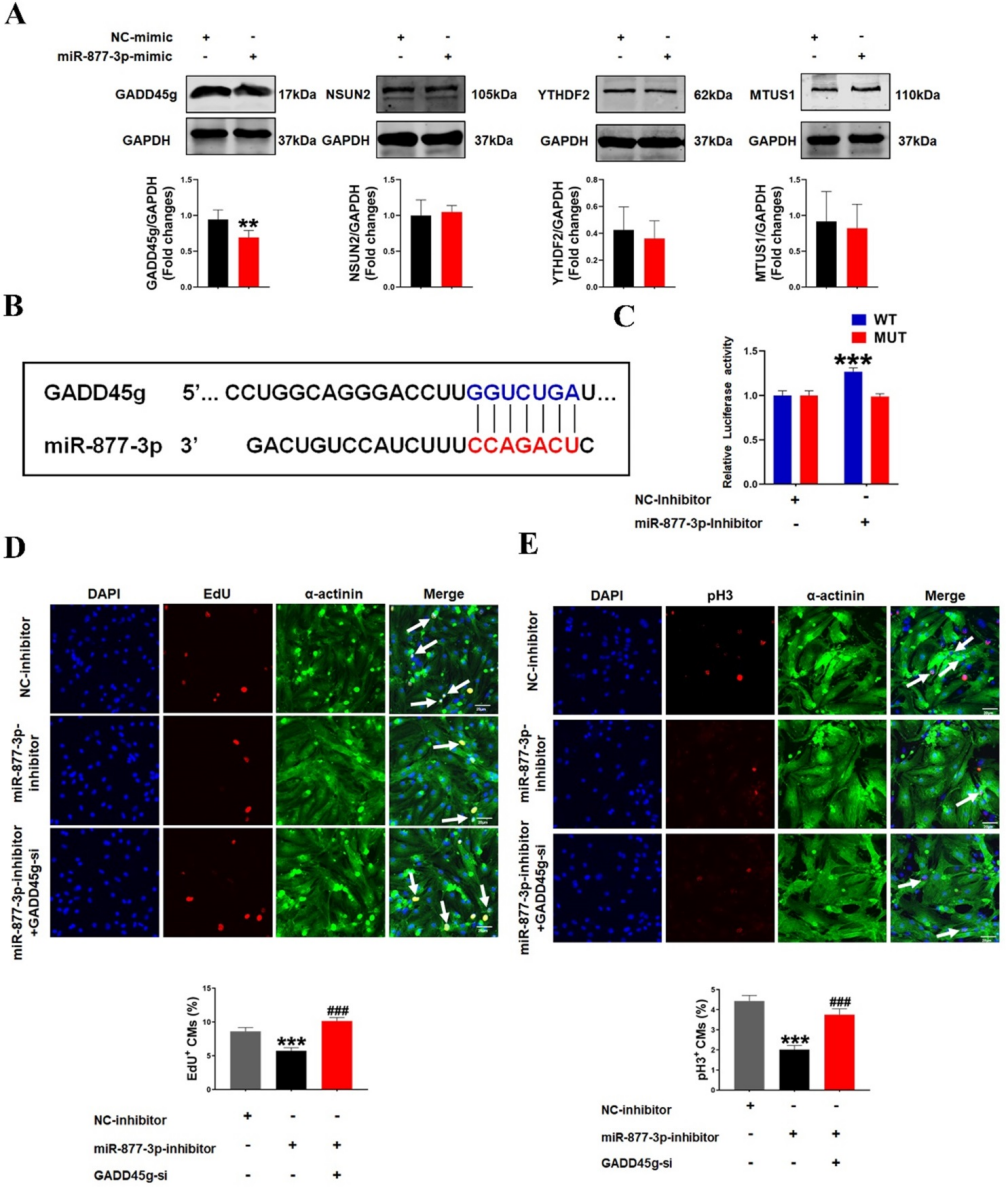
** MiR-877-3p regulates cardiomyocyte proliferation through targeting GADD45g. (A)** GADD45g, NSUN2, YTHDF2 and MTUS1 protein levels in CMs with miR-877-3p upregulation were determined by Western Blot. **P < 0.01 vs. NC-mimic. **(B and C)** The luciferase assay was used to analyze the binding potential between miR-877-3p and GADD45g. **(D and E)** CMs proliferative ability was evaluated by EdU and pH3 staining. Scale bar = 20 µm. ***P < 0.001 vs. NC-inhibitor, ^###^*P* < 0.001 vs. miR-877-3p-inhibitor. The data represent the mean ± SEM.

To investigate how miR-877-3p affects the proliferative effects of LED-Red on CMs, we further explored the potential target genes of miR-877-3p in neonatal CMs. By searching for the potential targets of miR-877-3p, we found that among the potential targets regulated by miR-877-3p, GADD45g, NSUN2, YTHDF2 and MTUS1 have regulatory roles in the involvement of cardiovascular disease 30-32 (Figure S1). We first detected the protein expressions of these four target genes after overexpression of miR-877-3p by Western Blot. The results showed that GADD45g protein levels were suppressed after miR-877-3p overexpression, whereas NSUN2, YTHDF2 and MTUS1 were not altered (Figure 3A). Members of the Gadd45 protein family are induced by DNA damage and other stress signals associated with growth arrest and apoptosis 34. In addition, inhibition of GADD45g expression reduces apoptosis in neonatal CMs in myocardial ischemia-reperfusion 30. In this study, we focused on exploring the relationship between miR-877-3p and its target gene GADD45g. We found that miR-877-3p has a binding site on the target gene GADD45g (Figure 3B). And the luciferase assay further confirmed that miR-877-3p may target at the 3'UTR of GADD45g (Figure 3C). This indicates that GADD45g acts as a direct target gene of miR-877-3p.

**Figure 4:**
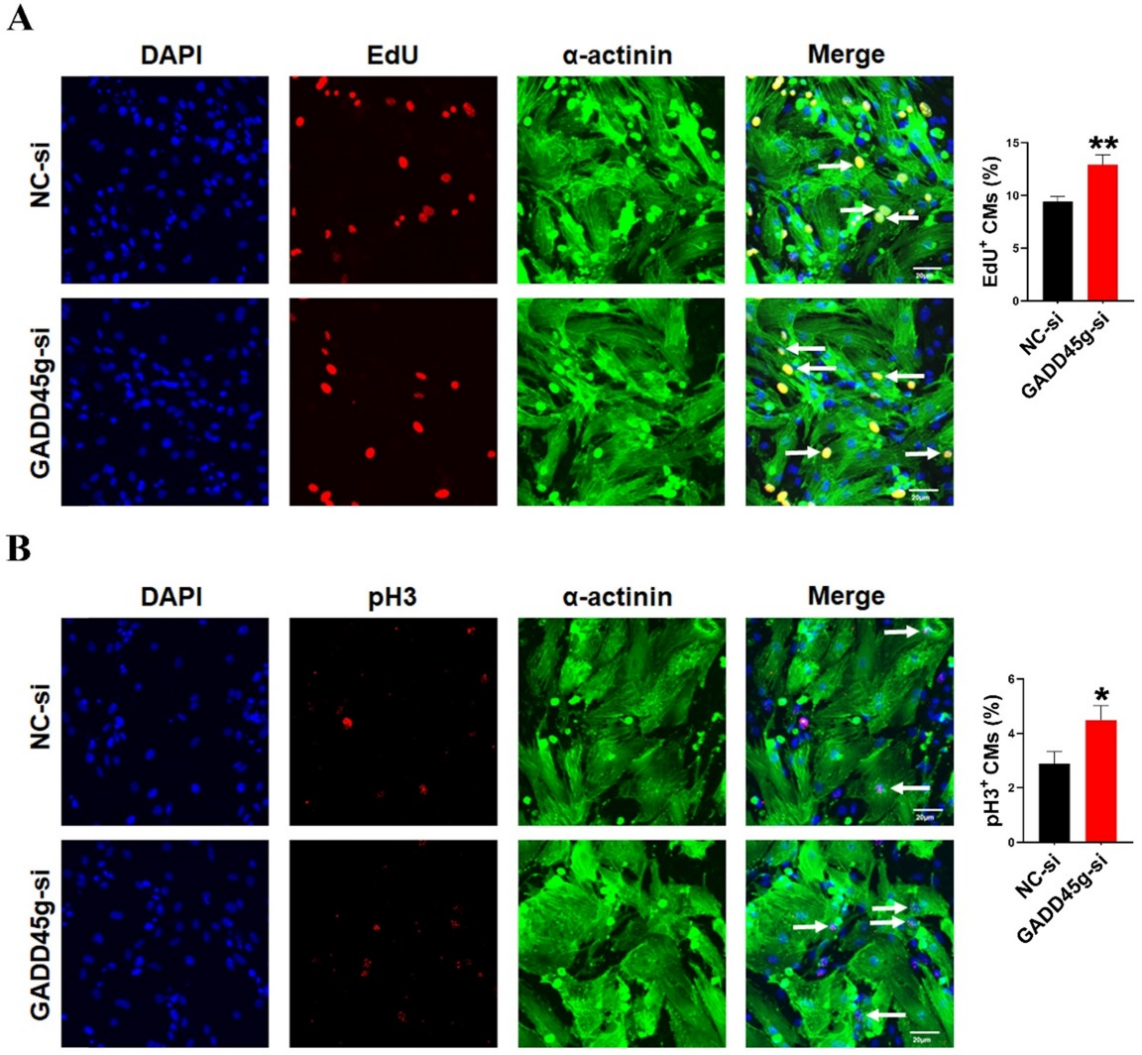
** Inhibition of GADD45g expression promotes cardiomyocyte proliferation.** CMs were transfected with GADD45g-si and immunofluorescence of **(A)** EdU and **(B)** pH3 staining were performed to detect CMs proliferation. *P < 0.05, **P < 0.01 vs. NC-si. The data represent the mean ± SEM. Scale bar = 20 µm.

In order to further confirm the role of GADD45g in miR-877-3p induced proliferation of neonatal CMs, we knocked down the expression of GADD45g by its siRNA, and exposed CMs to miR-877-3p inhibitor. Then, we observed the effects of GADD45g silencing on the proliferative of neonatal CMs. As shown in Figure 3D and 3E, the inhibition of neonatal CMs proliferation effect by miR-877-3p knockdown was also abolished after concomitant inhibition of GADD45g expression. More importantly, our result showed an elevated proliferation rate of neonatal CMs after GADD45g-si treatment of cells alone, compared to the control group. This suggests that GADD45g produces a regulatory effect on the proliferation of neonatal CMs (Figure 4A, B). This result supports GADD45g as a target gene of miR-877-3p to mediate the proliferation of neonatal CMs.

### LED-Red targets GADD45g to promote the proliferation of cardiomyocytes

**Figure 5:**
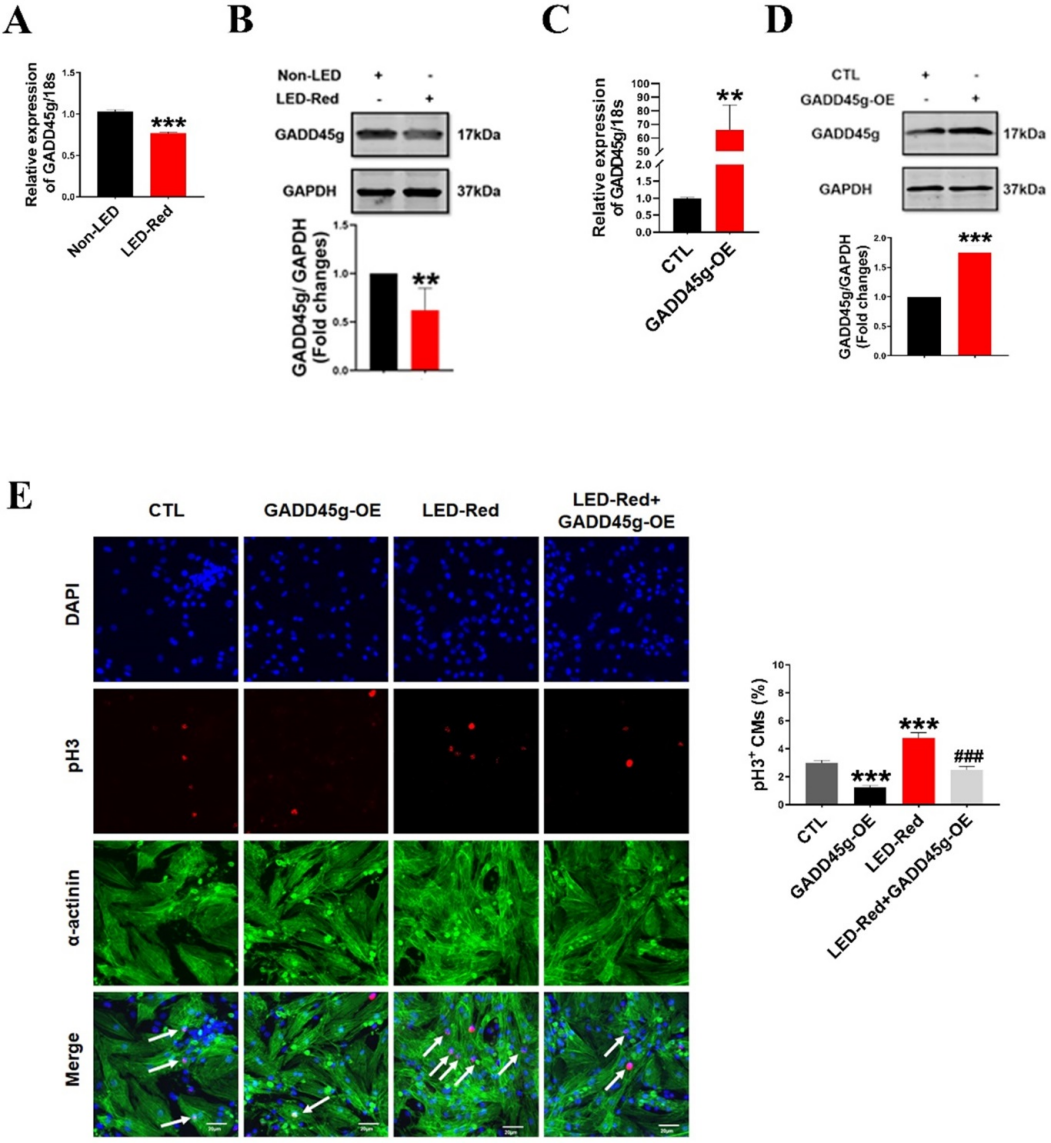
** LED-Red regulates GADD45g to promote the proliferation of cardiomyocytes. (A and C)** qRT-PCR was used to assay the RNA expression level of GADD45g in CMs. **(B and D)** Western Blot was used to verify GADD45g protein levels in CMs. **(E)** Effect of LED-Red irradiation and co-treatment with GADD45g overexpression on CMs proliferation were verified by pH3 staining. Scale bar = 20 µm. The data represent the mean ± SEM. *P < 0.05, **P < 0.01, ***P < 0.001 vs. CTL,^ ###^*P* < 0.001vs. LED-Red group.

To determine whether the GADD45g is also the downstream target gene of LED-Red, we observed the changes of mRNA and protein levels of GADD45g after LED-Red stimulation. The results showed that the mRNA expression of GADD45g was downregulated in neonatal CMs after LED-Red illumination (Figure 5A), consistent with protein expression of GADD45g (Figure 5B). Next, we verified the effect of LED-Red on the proliferation of neonatal CMs overexpressing the GADD45g plasmid. The overexpression of GADD45g in the neonatal CMs after transfection was firstly examined and verified (Figure 5C, D). Then, immunofluorescence pH3 staining showed that overexpression of GADD45g had an inhibitory role in the promotion of neonatal CMs proliferation by LED-Red (Figure 5E). This implies that GADD45g acts as a downstream of LED-Red in neonatal CMs.

### GADD45g acts as a downstream target of LED-Red/miR-877-3p in regulating cardiomyocyte proliferation

**Figure 6:**
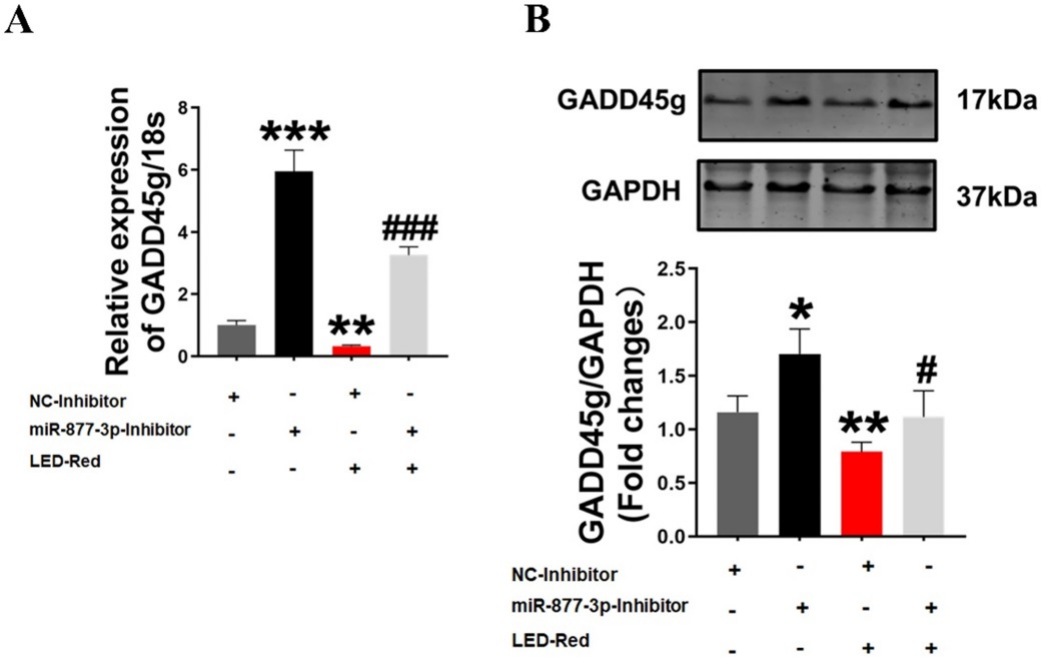
** GADD45g acts as a downstream target of LED-Red/miR-877-3p for regulating cardiomyocyte proliferation. (A)** qRT-PCR and **(B)** Western Blot were applied to verify the effect of LED-Red and miR-877-inhibitor combined treatment on GADD45g expression. The data represent the mean ± SEM. *P < 0.05, **P < 0.01, ***P < 0.001 vs. NC-inhibitor,^ #^*P* < 0.05, ^###^*P* < 0.001vs. LED-Red + NC-inhibitor group.

Finally, to further confirm the role of GADD45g in the regulation of neonatal CMs proliferation by LED-Red/miR-877, we combined the application of LED-Red and miR-877-3p inhibitor to treat neonatal CMs. The results showed that the mRNA expression of GADD45g inhibited by LED-Red was partially attenuated by miR-877-inhibitor (Figure 6A). More importantly, miR-877-inhibitor pretreatment abrogated the inhibitory effect of LED-Red on GADD45g protein expression in neonatal CMs (Figure 6B). These results indicate that LED-Red modulates the proliferation of neonatal CMs by regulating the expression of miR-877 and its downstream target GADD45g.

## Discussion

In the visible spectrum, red light owns a long wavelength and it easily penetrates tissues compared with other wavelengths of light 35, 36. It is often used clinically to treat tissue inflammation or some diseases, such as acne, phlebitis, tissue trauma, and scar repair 37. In addition, it has been suggested that LED-Red is classified as a “low energy laser” 36. PBM, as a novel biomedical technology, has been increasingly reported to be an adjuvant for the treatment of various diseases. However, the effect of PBM on the proliferation of CMs was still unclear. In the present study, we found that conditioned LED-Red activated the proliferation ability of neonatal CMs by upregulating miR-877-3p expression, which in turn prevented the expression of DNA damage gene GADD45g. Similarly, PBM has been shown to prolong the growth cycle of neural stem cells 25, 26. After receiving LED-Red irradiation or overexpression of miR-877-3p, the proliferation rate of neonatal CMs was up-regulated. Both LED-Red stimulation and miR-877-3p overexpression suppressed the protein levels of GADD45g in neonatal CMs. The proliferative ability of neonatal CMs was partially lost when they were treated with the combination of LED-Red and GADD45g overexpression. Likewise, previous study also showed that GADD45g played a negative role in the proliferation of dental epithelial cells 38.

The effectiveness of PBM therapy is not only related to the choice of wavelength, but also to the type of tissue disease. In antitumor therapy, blue light can act to kill cancer cells and inhibit their migration, but in neuronal cells it can exhibit a role in regulating the proliferation and differentiation of neural stem cells 19, 39. In another of our PBM studies, we compared the effects of various wavelengths in the visible spectrum on CMs viability. LED-Red was identified to significantly increase the viability of CMs compared to blue, green and yellow light. Therefore, red light was chosen as the main light source for PBM 27. MiRNAs have been reported to play a critical role in regulating key aspects of cardiac physiological and pathological signaling, including the regulation of cardiac regeneration *in vitro* and *in vivo*^40^. In a study by Feliciano* et al.*, PBM was found to exert a regulatory effect on transcription factors associated with apoptosis within myocardial tissue of rats after myocardial infarction 41. In addition, Allan *et al.* found that PBM reduced the expression of miR-24 in human-induced pluripotent stem cell-derived ventricular cardiomyocytes (hiPSC-vCMs) and reduced doxorubicin induced cardiotoxicity, indicating that PBM can act at the on post-transcriptional level 42. However, the relationship between PBM and miRNAs regulating CMs proliferation has not been reported. In the present study, we revealed that LED-Red irradiation could regulate the level of miRNAs and the expression of their downstream targets, which thus regulates CMs proliferation.

Previous studies have identified an important role of GADD45g in the molecular mechanism of anti-apoptotic myocardial death 30. Recent clinical researches have shown that GADD45g expression is elevated in various CMs and may serve as a biomarker of myocardial injury 43. In addition, GADD45g plays a role in regulating proliferation in different cancers 44. It was found that Gadd45 protein interacts with cyclin-dependent kinase 1 (Cdk1), which is a strong inhibitor of Cdk1-cyclin B1 activity, both* in vivo* and* in vitro* 45. Gadd45g also interacts with the proliferating cell nuclear antigen PCNA, a NER component that plays a role in DNA synthesis 46. The above reports potentially suggest that GADD45 protein is closely related to cell proliferation. In this study, we found that GADD45g knockdown can promote CMs proliferation, demonstrating that GADD45g could be served as a new target for regulating CMs proliferation. However, the regulation of living organism by light is a complicated process. The miR-877-3p/GADD45g axis is not the only mechanism by which LED-Red promotes CMs proliferation. And, other pathways of PBM involved in the regulation of myocardial proliferation still deserve future studies.

PBM, as a new type of biological therapy, has limitations in the applicability of its light source in biological organisms. In particular, the application of PBM to the heart requires consideration of the human physiology and the penetration of the light source. Some ideas for further research have arisen regarding the above issues. For example, treatment by injecting upconversion nanoparticles (UCNPs) into the heart in advance and irradiating it with a strong penetrating transcranial near-infrared light (NIR), as well as converting the NIR into a therapeutic wavelength in the visible range by conversion of UCNPs seems to be a good option 19, 47.

In summary, this study found that conditioned LED-Red could activate the proliferation ability of CMs. LED-Red exerted a pro-proliferative effect by increasing miR-877-3p and inhibiting the expression of GADD45g. These findings suggest that LED-Red as a potential biotechnology to regulate the proliferation of CMs, which might become a new non-invasive controllable biotechnology for the treatment of cardiovascular diseases.

## Supplementary Material

Supplementary figure and table.

## Figures and Tables

**Figure 7:**
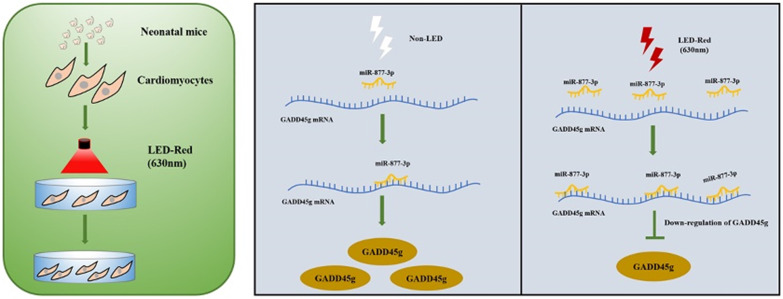
** A Scheme Model for the Role of LED-Red/miR-877-3p/GADD45g axis in cardiomyocyte proliferation.** A programmatic model of LED-Red regulation of miR-877-3p and the downstream target gene GADD45g in CMs proliferation.

## Abbreviations

PBM: Photobiomodulation
miRNAs: microRNAs
CMs: cardiomyocytes
LEDs: Light-emitting diodes
LED-Red: 630nm Light Emitting Diode
pH3: phospho-Histone H3
EdU: Ethynyl-2deoxyuridine incorporation
