# Supplementary material for: Light Emitting Diodes Irradiation Regulates miRNA-877-3p to Promote Cardiomyocyte Proliferation
Source: Int J Med Sci. 2022 Jul 11;19(8):1254–64. doi: 10.7150/ijms.70743 (PMC9346386; doi:10.7150/ijms.70743)
Supplement: Supplementary file 1 — Supplementary figure and table. [file ijmsv19p1254s1.pdf]

Supplementary Figure 1. Sequences of miR-877-3p binding sites to NSUN2, YTHDF2 and MTUSI.

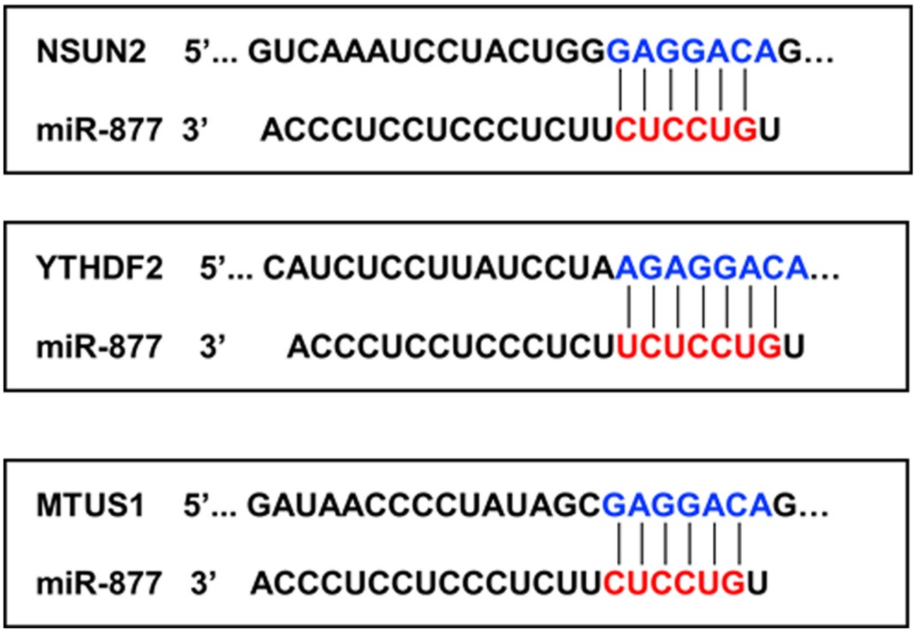

Table S1.

GENE	*Forward primer*	*Reverse primer*
miR-590-3p	GGCCTAATTTTATGTATAAGCTAGT	ATCCAGTGCAGGGTCCGAGG
miR-15b-3p	CGAATCATTATTTGCTGCTCTA	ATCCAGTGCAGGGTCCGAGG
miR-19b-3p	TGTGCAAATCCATGCAAAACTGA	ATCCAGTGCAGGGTCCGAGG
miR-143-3p	CCGCCCTGAGATGAAGCACTG	ATCCAGTGCAGGGTCCGAGG
miR-877-3p	TGTCCTCTTCTCCCTC	TGTCGTGGAGTCGGC
miR-877-3p mimics	UGUCCUCUUCUCCCUCCUCCCA	GGAGGAGGGAGAAGAGGACA
miR-877-3p inhibitor	UGGGAGGAGGGAGAAGAGGACA	
Gadd45g siRNA	CCGCCAAAGUCCUGAAUGUTT	ACAUUCAGGACUUUGGCGGTT
U6	GCTTCGGCAGCACATATACTAAAAT	CGCTTCACGAATTTGCGTGTCAT
18s	CATTCGAACGTCTGCCCTATC	CCTGCTGCCTTCCTTGGA
